# Songs, Stories, Design, and Dialogue: Intervention Mapping, Human- Centered Design, and Arts-Based Methods for a Chhaupadi (Menstrual Seclusion) Intervention Development in Nepal

**DOI:** 10.21203/rs.3.rs-10525651/v1

**Published:** 2026-10-13

**Authors:** Sara Baumann, Sanjana Murthy, Nikita Sharma, Annika Agarwal, Sudha Ghimire, Aubree E. McMahon, Bhimsen Devkota, Elizabeth Miller, Hannah Schwarz, Ada Youk, Sara Parker, Mary Hawk

## Abstract

**Background:** Menstrual stigma and chhaupadi (menstrual seclusion) persist in rural Nepal despite criminalization in 2017, highlighting the limitations of legal approaches and interventions implemented to date. Creative and community-engaged tools such as arts-based methodologies and human-centered design (HCD) offer participatory, iterative approaches that center community voices in intervention development. However, its application to menstrual health in rural Himalayan settings remains largely unexplored. Pairing creative methods with a structured approach – Intervention Mapping (IM) – allows for a flexible yet rigorous participatory and evidence-based approach for designing solutions to address complex health issues. **Methods:** Eleven women currently practicing chhaupadi were recruited for heterogeneity to participate in a 10-day co-design workshop in Dailekh, Nepal. Participants engaged with a package of HCD and arts-based tools, including collaborative storytelling, photo journaling, clay modeling, role-play, participatory art, deuda folk song composition, and more. These activities were guided by the IM framework, including a needs assessment, developing change objectives, and program design activities. Concurrent Key Informant Interviews and Community Dialogue Sessions added insights. Data presented in this paper was generated through facilitator field notes, team debriefs, workshop documentation, and post-workshop reflections with participants. **Results:** Culturally grounded and multimodal tools generated the highest levels of engagement and meaning making, enabling participants to participate as active co-designers. Relational practices, like Walk-a-Mile home visits and culturally meaningful gestures of recognition functioned as key components of participation rather than supplementary activities. Notably, some participants began changing their behavior during the workshop itself, before any formal intervention was deployed, suggesting that the boundary between intervention development and intervention delivery is not as clear-cut as often assumed. Key challenges included cognitive demands of abstract tools such as the Impact Ladder, social tensions arising from participant selection, logistical constraints, and unmet childcare and economic needs. **Conclusions:** Integrating IM, HCD and arts-based methods demonstrate meaningful potential for menstrual health intervention development in Nepal. The co-design process itself functioned as an early mechanism of change, challenging the assumption that intervention development and behavior change are temporally distinct. Future co-design frameworks should build relational infrastructure into the research scaffolding from the outset.

